# Using the Gut Microbiome to Assess Stocking Efforts of the Endangered Pallid Sturgeon, *Scaphirhynchus albus*

**DOI:** 10.3390/life13020309

**Published:** 2023-01-22

**Authors:** Sarah Gaughan, John A. Kyndt, Justin D. Haas, Kirk D. Steffensen, Patrick M. Kočovský, Kevin L. Pope

**Affiliations:** 1 College of Science and Technology, Bellevue University, Bellevue, NE 68005, USA; 2 Nebraska Game and Parks Commission, Lincoln, NE 68501, USA; 3 U.S. Geological Survey, Reston, VA 20170, USA; 4 U.S. Geological Survey—Nebraska Cooperative Fish and Wildlife Research Unit, School of Natural Resources, Lincoln, NE 68583, USA

**Keywords:** gut microbiome, Missouri river, *Scaphirhynchus albus*

## Abstract

The endangered Pallid Sturgeon, *Scaphirhynchus albus*, has been actively managed to prevent population declines, including stocking of hatchery-raised fish. The gut microbiome plays an innate role in an organism’s absorption of nutrients by increasing nutrient availability and can provide new insights for Pallid Sturgeon management. In this study, the Pallid Sturgeon’s microbiome is dominated by the phyla Proteobacteria, Firmicutes, Actinobacteria and Fusobacteria. It was also determined that the gut bacterial diversity in hatchery-raised Pallid Sturgeon was not significantly different from wild Pallid Sturgeon, supporting that hatchery-raised Pallid Sturgeon are transitioning effectively to wild diets. There is also a high degree of intraspecific variation in the bacterial and eukaryotic sequences amongst individual Pallid Sturgeon microbiomes, suggesting the Pallid Sturgeon may be omnivorous. This study demonstrated that genetic markers may be used to effectively describe the dietary requirements for wild Pallid Sturgeon and provides the first genetic evidence that Pallid Sturgeons are effectively transitioning from hatchery-raised environments to the wild.

## 1. Introduction

The gut microbiome is a collection of microorganisms housed within an organism’s intestinal tract. The study of the microbiome has advanced significantly with genomic sequencing, with the first gut bacteria being sequenced in 1996 [1]. The gut microbiome plays integral roles in the host organisms’ absorption of nutrients through digestion and in organisms’ innate immunity [2,3]. 

Fish intestines, in particular, harbor diverse populations of microorganisms, especially bacteria [4,5]. Recent analysis of gut microbiome data of >200 fish species from Korea indicates that the possible diversity in fish gut microbiomes is much wider than in other studied animal species [6]. Fish microbiomes tend to be dominated by members of the phyla Proteobacteria, Firmicutes, Bacteroidetes, Actinobacteria and Fusobacteria [6,7]. There are three major groups of factors that dictate fishes’ gut microbiomes, including ecological conditions, environmental conditions and host trophic-level feeding habits [8]. Fluctuations in any of these groups of factors, such as transitions between environments, particularly if these environments have different feed availability, are likely to result in changes within the intestinal microbiome community structure [7,9,10,11]. Gender may also play a lesser role in the diversity of the microbiome [12,13]. Understanding how ecological conditions, environmental conditions and host trophic-level feeding habits impact a fish’s gut microbiome may offer new insights into conservation strategies and behaviors for fishes.

The endangered Pallid Sturgeon, *Scaphirhynchus albus*, is a native species of the Mississippi and Missouri Rivers [14,15], where stocking has been employed to bolster wild populations [16]. Hatchery supplementation has stocked 444,438 Pallid Sturgeon in the lower Missouri River from Gavins Point Dam to its confluence with the Mississippi River [17]. The population currently consists of 75% stocked Pallid Sturgeon [18]. Understanding gut microbiome composition and factors that assess the gut microbiome composition may facilitate stocking efforts for the endangered Pallid Sturgeon; however, the gut microbiome of Pallid Sturgeon remains unexplored.

Wild Pallid Sturgeons have been previously noted to consume Trichoptera, Ephemeroptera and Diptera [19,20]; however, recent work suggests their diet may be more varied [21]. Gut microbial communities of captive fishes can differ substantially from those of wild fishes due to prepared diet and increased density of organisms in a confined environment [22]. The diet fed to fish raised in a hatchery setting is a major driver that establishes an organism’s principal core microbiome. The core microbiome is the microbial taxa shared among two or more samples from a particular host or environment [23]. This diet may not adequately reflect the diet of a wild organism and, as such, this core microbiome may not adequately prepare this organism for release into the wild.

This study has two major goals. The first goal is to determine the core microbiome of the endangered Pallid Sturgeon. Studies on the gut microbiome of fish species have been largely limited to a few model species, such as zebrafish [24], guppy [7] and rainbow trout [25], and in economically valuable species, such as Atlantic salmon [26], Atlantic cod [27], sturgeon [28], carp [29] and tench [30]. Understanding the factors that impact the Pallid Sturgeon’s gut microbiome may facilitate conservation efforts. The second goal of this study is to provide a comparative analysis of the gut microbiome between hatchery-raised Pallid Sturgeons that were then released into the wild, compared to wild-raised species. Comparing intestinal microbial diversity and composition between hatchery-raised and wild Pallid Sturgeons may provide insight on the transition from the hatchery environment to a wild environment. In this study, (a) the gut microbiomes of hatchery-raised and wild Pallid Sturgeons was characterized, (b) the bacterial diversity between hatchery-raised and wild Pallid Sturgeon was compared and (c) other factors that could potentially impact Pallid Sturgeon microbiome diversity were explored. 

## 2. Materials and Methods

### 2.1. Sample Collection and DNA Sequencing 

Pallid Sturgeons for gut microbiome sampling were collected by Nebraska Game and Parks Commission biologists during routine broodstock sampling that is part of Nebraska’s sturgeon management initiative (Table 1). Pallid Sturgeons were collected with stationary trotlines in a 24 km reach of the Missouri River from 4 to 13 April 2018. Pallid Sturgeons were classified as wild or hatchery-reared based on hatchery marks (i.e., scute removal marks, passive integrated transponder, code wire tag or various combinations) or genetic analysis [18].

Colonic samples were obtained from Pallid Sturgeons using colonic flushing. The flushing apparatus consisted of a 60 mL catheter-tip syringe (#CTSLS3, Care Touch, Brooklyn, Brooklyn, New York, NY, USA) fitted with a 41 cm, 3.3 mm urethral catheter commonly used in veterinary medicine (#701017, Kendall Company, Mansfield, MA, USA). The bottle was filled with distilled water and the catheter end was gently inserted 30–50 mm through the fish’s anus into the colon. The colon was flushed until expelled water was clear. All materials (solid feces, flushed liquid) were poured into a 500 mL sample jar and preserved with an equal volume of 100% ethanol. 

Total DNA was extracted using the MoBio PowerSoil DNA Isolation Kit (MoBio, Carlsbad, CA USA). The primer pair used for amplification of the V3-V4 region of the 16S amplicon was S-D-Bact-0341-b-S-17 and S-D-Bact-0785-a-A-21 [31]. Primers were added according to the TaggiMatrix 16S PCR Protocol using fusion-indexed primers [32]. The V3-V4 region of the 16S ribosomal RNA gene was sequenced with an Illumina MiSeq on a PE300 run at the Environmental Health Science laboratory at the University of Georgia. Sequences were trimmed with GENEIOUS™ 10.2.6 using default parameters (Biomatters, Newark, NJ, USA) after raw reads were paired. Chimeric reads were removed following merging of paired reads and were analyzed using the metagenomic 16S analysis pipeline in GENEIOUS™ 10.2.6 with default parameters (Biomatters, Newark, NJ, USA) [33]. All raw sequencing reads were deposited into NCBI (PRJNA806960).

### 2.2. Core Gut Microbiome

Bacteria that comprise the five highest relative abundances were considered part of the Pallid Sturgeons’ core gut microbiome. Relative abundance was calculated as a percentage of the consensus regions of the 16S gene of an identified bacterial genus divided by the total number of 16S gene sequences [34]. The average relative abundance of 16S reads had to be greater than or equal to 0.1% at the genus rank to be included in the statistical analysis. 

### 2.3. Comparison between Hatchery-Raised and Wild Pallid Sturgeon Gut Microbiomes

A Kruskal–Wallis test with a Bonferroni correction to minimize Type I error [35,36] was used to determine the effect of origin (hatchery-raised or wild) on the relative abundance of bacteria within the gut bacterial microbiome. The Kruskal–Wallis test with a Bonferroni correction was conducted in R Studio Version 1.1.463. Statistical significance was set at 0.05.

Diversity is a measure of number, type and/or evenness of bacteria within the gut of the Pallid Sturgeon. Alpha diversity is the average bacterial diversity in the gut bacterial microbiome of each Pallid Sturgeon and was calculated using the Shannon–Wiener diversity index [37]. Alpha diversity was calculated after square-root transformation using PRIMER-e Version 7 (Quest Research Ltd., Auckland, New Zealand [35]). Average *H* was used to compare the alpha diversity of gut bacterial microbiomes of hatchery-raised Pallid Sturgeons to the alpha diversity of gut bacterial microbiomes of wild Pallid Sturgeons at both the phylum level and the genus level. An independent sample t-test analysis was conducted in R Studio Version 1.1.463 to assess the differences in alpha diversity of the gut bacterial microbiome in relation to the origin of Pallid Sturgeons (hatchery-raised vs. wild) at both the phylum level and the genus level, with statistical significance set at 0.05. An ANOVA was conducted in R Studio Version 1.1.463 to assess the origin of the Pallid Sturgeon (hatchery-raised vs. wild) in relation to collection location at both the phylum level and the genus level (four analyses total). Statistical significance was set at 0.05.

Beta diversity is the dissimilarity between the microbiomes of each Pallid Sturgeon and was calculated using PRIMER-e Version 7 (Quest Research Ltd., Auckland, New Zealand [35]) using the Sørensen similarity index, as described by Diserud and Odegaard [38]. Beta diversity was calculated using the betapart package [39] in R Studio Version 1.1.463. An independent sample t-test analysis was conducted in R Studio Version 1.1.463 to assess the differences in beta diversity of the gut bacterial microbiome in relation to the origin of the Pallid Sturgeon (hatchery-raised vs. wild) at both the phylum level and the genus level with a statistical significance set at 0.05. An ANOVA was conducted in R Studio Version 1.1.463 to assess the origin of the Pallid Sturgeon (hatchery-raised vs. wild) in relation to collection location, at both the phylum level and the genus level (four analyses total). Statistical significance was set at 0.05.

Principal Coordinate Analysis (PCoA) ordination plots, heat maps and stacked bar plots comparing all samples were generated in MG-RAST (version 4.0) [40]. For MG-RAST analyses, the raw data were preprocessed in the MG-RAST pipeline. After being uploaded to MG-RAST, data are preprocessed by using SolexaQA [41] to trim low-quality regions (Phred quality score, Q < 13) from FASTQ data. Potential human sequencing reads were removed using Bowtie [42] (a fast, memory-efficient, short read aligner), and only filtered sequences passed into the next stage of the annotation pipeline. A BLAST similarity search for the longest rRNA cluster representative is performed against the M5rna database, which integrates SILVA [43], Greengenes [44] and RDP [45]. MG-RAST uses the R package DEseq for normalization. The thresholds for the stacked bar plot in MG-RAST were set at e-value: 5; percent identity: 70; minimal alignment length: 15; min. abundance: 100 for phylum analysis, and e-value: 5; percent identity: 90; minimal alignment length: 15; min. abundance: 2000 for genus analysis. Bray–Curtis distance method with normalization was used to generate the PCoA ordination plots [46]. Principle coordinate 1 and 2 variations were 0.6967 and 0.1325, respectively, for the phylum analysis and 0.6962 and 0.06499 for the genus analysis. 

## 3. Results

### 3.1. Pallid Sturgeon Gut Microbiome

Total DNA was extracted from 44 fish (Table 1), 34 that were hatchery-raised and 7 that were reared in the wild, as well as 3 of unknown origin, from five sites along the Missouri River (Table 1). Two of the hatchery samples (tags 434A5C7340 and 43693D7261) did not generate sufficient DNA for sequencing; therefore, 42 samples, in total, were used for sequencing and further analysis. In total, 1,995,012 raw reads were obtained for both forward and reverse directions, with a depth of 1,929,957 ± 96,497 sequences. After quality filtering and merging trimmed paired reads, 84,740 reads were mapped to exons with a mean of 1970 reads/sample. 

**Figure 1:**
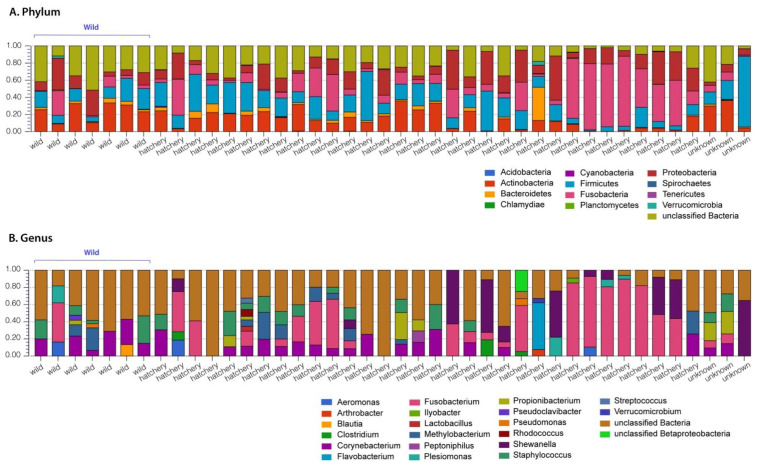
Stacked bar plot overview of the microbial composition of all samples at the phylum level (**A**) and the genus level (**B**). Annotation was performed using the SILVA SSU database in MG-RAST. Stacked bars were normalized and the original fish habitat is indicated on the X axis.

Alpha diversities ranged from 1.81 to 3.78 for Pallid Sturgeons. Beta diversity in the gut bacterial microbiome of Pallid Sturgeons ranged from 0.6 to 0.84. Stacked bar plots comparing the phylum- and genus-level microbial composition (Figure 1) show that there is a large amount of intraspecific variation the microbiomes observed. Overall, at the phylum taxonomic rank, the microbiome was dominated by Fusobacteria, Firmicutes, Proteobacteria, Actinobacteria and Bacteriodetes. 

**Figure 2:**
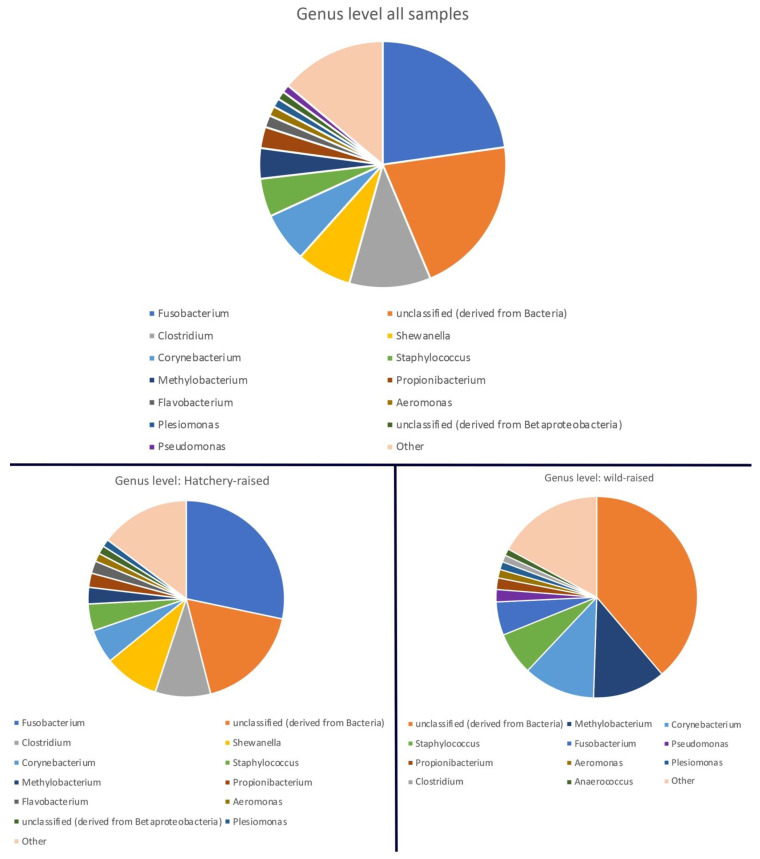
Overview of the genus-level composition of the Pallid Sturgeon core microbiome of all samples combined (**top**), and microbial composition of the hatchery-raised (34) and wild-raised (7) samples separately (**bottom**). Percentages of genus representations were calculated in MG-RAST. Only genera with representations >1% are shown and the rest are combined as ‘other’ for clarity.

Figure 2 shows a genus-level overview of the bacterial composition of all samples combined. In total, 321 bacterial genera were identified at relative abundances ≥ 0.1%. Only genera with representations > 1% are shown in Figure 2 for clarity. At the genus taxonomic rank, the core bacterial gut microbiome was dominated by *Fusobacterium, Clostridium, Corynebacterium, Shewanella, Staphylococcus*, *Flavobacterium*, *Propionibacterium, Aeromonas* and *Methylobacterium*. When comparing the genus-level microbiome of the hatchery-raised versus wild-raised samples, the representation of the major genera appears to be very similar (Figure 2 bottom). Although the wild-raised samples appear to contain lower levels of *Shewanella* and *Clostridium*, and a higher level of *Methylobacterium* and unclassified bacteria, the wild-raised samples only compromised 7 samples while there were 34 hatchery-raised samples and overall diversity and multidimensional analyses are needed to determine any potential significant differences (see below). The level of ‘unclassified Bacteria’ of ~20% at the genus level is not unsurprising, given the fact that this analysis is using environmental samples of a newly studied species and many environmental bacterial microbes have not been identified yet. 

### 3.2. Comparison between Hatchery-Raised and Wild Pallid Sturgeon Gut Microbiomes

Alpha diversity was 2.87 (range 1.81–3.78) for hatchery Pallid Sturgeons and 3.2 (range 3.14–3.29) for wild Pallid Sturgeons. There were no significant differences in alpha diversity in bacterial genera of the gut microbiome using the average Shannon–Wiener diversity index (*H*) between hatchery-raised Pallid Sturgeon and wild Pallid Sturgeon (*p* = 0.16, degrees of freedom = 2). There was no significant difference between relative abundances of bacterial genera in guts of hatchery-raised Pallid Sturgeons compared to wild Pallid Sturgeons (*p* = 0.81, degrees of freedom = 2). All hatchery-raised fish lived in the wild for at least 7 years, which may contribute to the lack of a significant overall difference in the diversity or in the microbial composition of the gut microbiome between the hatchery and wild-raised fish.

Beta diversity in the gut bacterial microbiome of hatchery-raised Pallid Sturgeons was 0.60. Beta diversity in the gut bacterial microbiome of wild Pallid Sturgeons was 0.84. There was no significant difference in beta diversity between the bacterial phyla of the gut microbiome of hatchery-raised Pallid Sturgeons compared to the beta diversity in the bacterial phyla of the gut microbiome of wild Pallid Sturgeons (*p* = 0.14, degrees of freedom = 2). There was no significant difference in beta diversity in the bacterial phyla in relation to collection location (*p* = 0.12, degrees of freedom = 2). 

There was no significant difference in beta diversity between the bacterial genera of the gut microbiome of hatchery-raised Pallid Sturgeons compared to the beta diversity in the bacterial genera of the gut microbiome of wild Pallid Sturgeons (*p* = 0.10, degrees of freedom = 2). There was no significant difference in the beta diversity in the bacterial genera in relation to collection location (*p* = 0.11, degrees of freedom = 2). 

The stacked bar plots comparing the microbial composition (Figure 1) also shows that the wild gut microbiome samples are essentially indistinguishable from the composition of the hatchery-raised samples; however, substantial variation can be seen between the individual samples, where higher levels of Fusobacteria are found in some samples with a lower abundance of Firmicutes and Actinobacteria. Although only limited wild samples were obtained to compare (due to the endangered nature of the species), the higher Fusobacteria levels appear to occur in both hatchery and wild samples. As such, 12 hatchery samples and 1 wild sample have Fusobacteria levels > 30% (Figure 1). 

**Figure 3:**
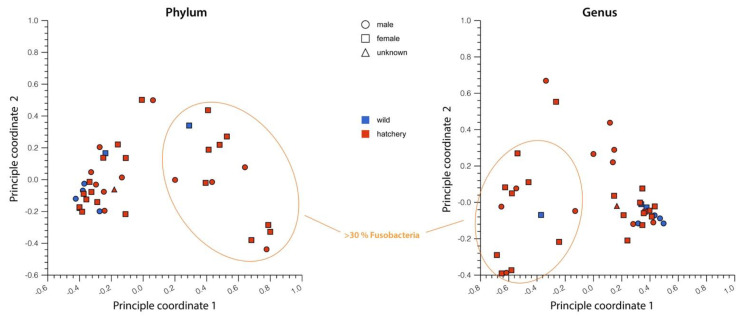
Principal Coordinate Analysis (PCoA) ordination plots comparing the overall bacterial gut microbiome composition of hatchery-raised (red) and wild Pallid Sturgeon (blue) at the phylum level (**left**) and the genus level (**right**). PCoA analysis was performed in MG-RAST. Microbiomes that contained 30% or more Fusobacteria are circled in orange.

To compare the multidimensional variation in the microbial composition of the gut microbiome samples, a Principal Coordinate Analysis (PCoA) of all samples was performed in MG-RAST at the phylum and the genus level (Figure 3). There was no clear separation between the wild and hatchery-raised samples at either the phylum- or the genus-level analyses, indicating that there is a shared microbiome.

### 3.3. Other Factors That May Affect Microbiome Variation

Except for one specimen, the sex of each fish was determined when sampling. Given the fact that the gut microbiome could be influenced by gender as in some other species, the microbiome was analyzed in function of gender. As can be seen in the PCoA plots in Figure 3, there was no significant difference in the microbiome composition in relation to sex. 

**Figure 4:**
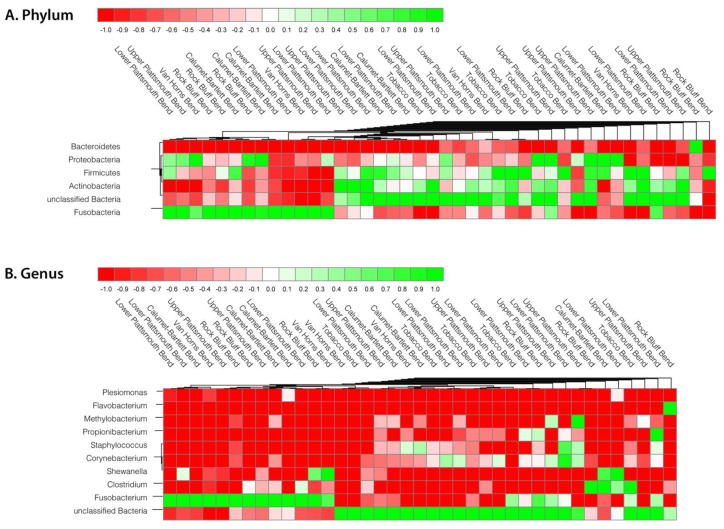
Heat maps and dendrograms of the microbial composition (**A**) at the phylum level (top-5 phyla and ‘unclassified’) and (**B**) at the genus level (top-10 genera) versus the sampling location.

The major phyla from the microbiome composition in relation to the sampling location were also analyzed (heat map representation in Figure 4). The analysis was performed at both the phylum and genus level. Higher-level *Fusobacterium* samples were found in the locations Lower Plattsmouth Bend, Upper Plattsmouth Bend, Van Horns Bend, Rock Bluff Bend, Calumet-Barlett Bend and in lower amounts in Tobacco Bend sampling locations (Figure 4) and, therefore, are not location specific. Further, at the phylum level, the higher levels of Fusobacteria versus Proteobacteria or Firmicute/Actinobacteria do not appear to be linked to a specific sampling location, as representatives of each of these are found in various locations. 

**Figure 5:**
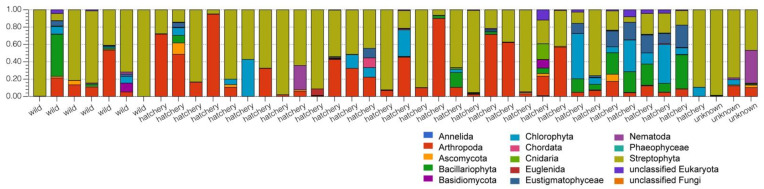
Stacked bar plot overview of the eukaryotic composition of all samples at the phylum level. Annotation was performed using the SILVA SSU database in MG-RAST. Stacked bars were normalized and the original fish habitat is indicated on the X-axis.

Although our experimental setup was not specifically targeting Eukaryote sequences, the SILVA SSU database analysis in MG-RAST allowed us to select for (low-abundance) eukaryotic signature sequences. The eukaryotic sequences represented between 0.1 and 6.4% of the reads in the samples using the SILVA SSU database comparison. There was a high amount of intraspecific variation observed in eukaryotic sequences present in the Pallid Sturgeon’s microbiome (Figure 5). The high variety of eukaryotic sequences observed in the intestinal microbiome suggests a wide array of potential diets.

## 4. Discussion

Gut microbiota are believed to play intrinsic roles in health, growth and disease status in animals [47,48,49]. Previous studies have demonstrated that fish can have complex gut microbiomes (both in the wild and captivity) that have taxonomic diversity similar to that of mammals [24,50]. Some factors that may affect fish microbiomes include life stage and ecological factors [24,50,51,52,53,54]. 

Maintaining breeding populations of animals in captivity, including the endangered Pallid Sturgeon, may well require managing their gut microbiomes [55]. In order to manage the gut microbiomes, more research must be conducted to establish what the ’ideal’ microbiome would be for each species and what factors may impact the composition or function of the microbiome. Typically, a healthy gut microbiome in animals is characterized by large bacterial taxonomic diversity, whereas a loss of diversity and expansion of more pathogenic bacterial species are typically associated with increased frailty and disease [56,57]. In our study, the gut microbial composition of hatchery-raised pallid sturgeons that were released in the wild overlap with the bacterial composition in the wild sturgeon and was contained within the natural variation in the species (Figure 3, PCoA plots). Based on this, the Pallid Sturgeon’s microbiome can be described, irrespective of whether they were raised in hatchery conditions or in the wild. Both the wild and hatchery-raised gut microbiomes are dominated by the phyla Proteobacteria, Firmicutes, Actinobacteria and Fusobacteria (Figure 1 and Figure 2). These phyla have dominated other fishes’ microbiomes and may represent a ‘core microbiome’ [6,58,59]. 

There is a small group of divergent samples evident in the PCoA analysis with a higher abundance (>30%) of Fusobacteria in those samples (Figure 3). With the exception of one wild microbiome sample, these are all hatchery-raised samples. The higher level of Fusobacteria was not found to be correlated with any of the different recorded ecological factors, including location, sex, weight and body composition, and age (only recorded for 16 samples). All of the identified Fusobacteria in our study belong to the genus *Fusobacterium*. Strains of *Fusobacterium* are generally considered pathogens to humans and cause several human diseases, including periodontal diseases, Lemierre’s syndrome and topical skin ulcers, and are found in higher levels in humans with colon cancer [60,61,62,63]. Although older reports might indicate Fusobacteria as gut commensal bacteria associated with healthy patients, these strains were reclassified as *Faecalibacterium* (Clostridiales: Ruminococcaceae) in 2002 [64]. On the other hand, studies of the gut microbiota of channel catfish *Ictalurus punctatus*, largemouth bass *Micropterus salmoides* and bluegill *Lepomis macrochirus* have indicated high levels of Fusobacteria (mainly the genus *Cetobacterium*), suggesting that these species might be part of the common gut microbiome of fish species [65]. Bacterial 16S rRNA amplicon sequencing does generally not have enough resolution to determine taxonomy at the species level, so further studies will be necessary to determine whether these Pallid Sturgeon *Fusobacterium* species are pathogens or part of the common fish gut microbiome (similar to the related *Cetobacterium*). 

Although there was no significant overall difference in the diversity or in the microbial composition of the gut microbiome between the hatchery and wild-raised fish, which may be because our hatchery-raised fish have been living in the wild for at least 7 years, there is substantial intraspecific variation in bacterial phyla and genera amongst individual samples (Figure 1). For example, in the hatchery-raised samples, the Proteobacteria varies between 3 and 46%, Firmicutes varies from 1 to 44%, Actinobacteria from 0.3 to 36% and Fusobacteria varies between 2 and 82%. Further, in the wild-raised samples, there are similar variations: Proteobacteria 5–35%, Firmicutes 6–28% and Fusobacteria 0.1–30%. The small N for wild fish may have made the gut diversity seem lower than it was or may have contributed to the lower proportion of wild fish within the >50% Fusobacterium group. Attempts to identify correlations between these phyla differences and the different recorded ecological factors, including location, sex, weight and body composition, and age, did not find any obvious correlations. This could be due to the small sample size or the lack of data on some of the variables. For example, the age of the specimen was only able to be confidently determined in 16 of the samples. The hatchery-raised species also originated from at least four different hatcheries and, for many specimens, the exact origin of the hatchery was unclear. These individual phylum- and genus-level variations are not correlated with the sampling location (Figure 4), which varied over a 24 km length of river. Therefore, at this point, the nature of the forces responsible for the individual variations in bacterial composition of the individual samples remains to be established. 

The main Proteobacteria genera found overall were *Aeromonas, Shewanella, Methylobacterium* and *Pleisiomonas*. The main genera from the Firmicutes were *Clostridium, Carnobacterium* and *Staphylococcus*, while *Corynebacterium* was the main Actinobacteria genus found. *Flavobacterium* was the main Bacteriodetes genus found. All of these were found in all samples, though in a wide range of varying ratios. Although *Aeromonas* has been found in several aquatic systems and fish gut microbiomes, it is generally regarded as a disease-causing pathogen of fish and is also linked to human infections [66,67,68]. On the other hand, based on enzymatic capabilities, both *Aeromonas* and *Staphylococcus* from the fish gut have been suggested to have positive effects on the digestive process of fish [69,70]. Clearly, further studies are needed to understand the complex interaction between the microbial species and the host, especially at the species level; however, *Clostridium* and *Shewanella* species have also been proposed as suitable candidates for probiotic treatments for fish gut microbiomes [70,71]. There is a growing interest in using probiotics as an alternative to antibiotic treatments in commercial aquaculture, which is currently a billion-dollar business. The development of so-called functional feed to improve the overall fish health and fish-farm productivity is a growing practice in modern aquaculture and, consequentially, the research on probiotics, prebiotics and immune stimulants has grown substantially in the last few decades [72,73,74,75]. A similar approach could be taken in directed conservation efforts, where fish populations (such as the Pallid Sturgeon) are provided with a feed that is optimized for the general gut microbiome of the specific species, before being released into the wild environment. As mentioned before, an initial baseline understanding of the gut microbiome diversity and compositions is essential to explore the impact of such future studies. 

Xu and Knight [76] suggested that long-term diet has the greatest effect on microbiome diversity. Fishes that were either herbivores or omnivores had a higher alpha diversity than fishes that were carnivores or piscivores [49]. Alpha diversity values calculated for the Pallid Sturgeon (2.80–3.33) fell in the upper range of alpha diversity values reported for other fishes [58,59], supporting recent research that the Pallid Sturgeon may be an omnivore [20,21] rather than a piscivore, as previously described [77]. Our preliminary screening of Eukaryotic signatures in our gut microbiome analysis (Figure 5) showed a wide variety of Eukaryota phyla, including arthropods, chordata, nematodes, plants and a variety of algal phyla, which would be consistent with an omnivore lifestyle. Future research should use alternative methods to identify prey items, such as sequencing the COX I gene, from colonic samples to test whether the Pallid Sturgeon is an omnivore [78].

Overall, there were no significant differences between alpha or beta diversities in gut bacterial microbiomes of hatchery-raised Pallid Sturgeon compared to wild Pallid Sturgeon, providing evidence that the gut microbiome of hatchery-raised Pallid Sturgeon transitioned effectively after release into the wild. Even so, the turnover process of gut microbiomes remains unexplored (assuming the gut microbiome was different in the hatchery environment). Intestinal epithelium turns over rapidly: approximately one to three billion and one-hundred to three-hundred million cells are shed per hour in the small intestine and in the colon, respectively [79]; therefore, gut microbiomes of the captured, hatchery-raised fish may have completed the transition in a brief period, perhaps days to weeks after release. Future research should look at different periods following release from the hatchery to explore how and when gut microbial transitions occur. 

Genetic markers provided a new way to assess management efforts for the endangered Pallid Sturgeon by facilitating new methods to explore the composition of their microbiomes. In this study, genetic markers demonstrated that the gut bacterial microbial diversity in hatchery-raised Pallid Sturgeon was not significantly different than wild Pallid Sturgeon; however, there is a large amount of intraspecific variation that may be due to diet. *Macrhybopsis* chubs were previously identified as a key prey item during the juvenile and adult stages for the Pallid Sturgeon, particularly the Shoal Chub, *Macrhybopsis hyostoma* [77]. Genetic markers from this study provide supporting evidence that Pallid Sturgeon may be omnivorous [20,21]. Sequencing additional genetic markers, such as the COX I gene [78], will help identify the breadth of the Pallid Sturgeon’s diet so that conservation managers can ensure Pallid Sturgeons have the resources they need to maintain a healthy body condition factor and aid future conservation efforts. 

Although this study showed that there is no significant difference in the gut microbiome of released versus wild-raised Pallid Sturgeons after several years, future research should look at Pallid Sturgeons’ microbiome prior to hatchery release and sample these individuals in subsequent years to determine how quickly microbial turnover occurs in relation to the wild environment. No correlation was observed with the sample location; however, such a fine-tuned time study might reveal some initial microbial differences that form a basis for later gut diversity patterns.

## Figures and Tables

**Table 1:** Geographic data for the collected Pallid Sturgeons.

Origin	Sex	Capture Location	Date Collected	Tag Number	Year Class	Yrs Since Released from Hatchery to 2018
Hatchery	Female	Calumet-Bartlett Bend: River km 937.8–931.5	2018/4/12	4627152F1A	2002	15
			2018/4/13	470A643317	2005	12
		Lower Plattsmouth Bend: River km 952.2–947.9	2018/4/4	4A466B2E78	2007	10
			2018/4/5	434771011A	2008	9
			2018/4/6	4900607F63	2007	10
			2018/4/7	434A03184E	2002	15
		Rock Bluff Bend: River km 943.1–937.8	2018/4/5	434A5C7340	2009	8
			2018/4/10	4627056D3B	2004	13
			2018/4/10	4627273324	2002	15
			2018/4/13	471269730D	2002	15
		Tobacco Bend: River km 947.9–943.1	2018/4/5	43693D7261	2006	11
			2018/4/6	4627313872	2003	14
			2018/4/11	47191F2B24	2006	11
		Upper Plattsmouth Bend: River km 956–952.2	2018/4/8	435F151E79	2002	15
			2018/4/8	43615C157E	2002	15
			2018/4/8	4367560D5D	2002	15
			2018/4/8	4369627915	2002	15
			2018/4/13	471E0C4B0E	2002	15
		Van Horns Bend: River km 927.6–924.7	2018/4/10	44451B466D	2002	15
			2018/4/10	46264C5368	2003	14
			2018/4/10	471979463C	2007	10
			2018/4/10	487F075D74	2009	8
Hatchery	Male	Calumet-Bartlett Bend: River km 937.8–931.5	2018/4/10	46267F6129	2006	11
			2018/4/12	4627702A4D	2009	8
		Upper Plattsmouth Bend: River km 956–952.2	2018/4/6	47161C0357	2002	15
			2018/4/6	47191A7D15	2008	10
			2018/4/6	847F623E77	2007	10
			2018/4/8	4A467F4F41	2007	10
		Rock Bluff Bend: River km 943.1–937.8	2018/4/5	434A41496C	2010	7
			2018/4/5	46280A267E	2005	12
			2018/4/10	46267A6226	2009	8
		Tobacco Bend: River km 947.9–943.1	2018/4/6	4349450B3E	2004	13
		Upper Plattsmouth Bend: River km 956–952.2	2018/4/7	434A4F216C	2008	9
Hatchery	Unknown	Tobacco Bend: River km 947.9–43.1	2018/4/6	4257361E77	2002	15
Unknown	Unknown	Lower Plattsmouth Bend: River km 952.2–947.9	2018/4/7	434A6B1F62		
		Upper Plattsmouth Bend: River km 956–952.2	2018/4/7	46256D3718		
Unknown	Male	Rock Bluff Bend: River km 943.1–937.8	2018/4/10	434969543E		
Wild	Female	Lower Plattsmouth Bend: River km 952.2–947.9	2018/4/4	4B191E7809		
		Upper Plattsmouth Bend: River km 956–952.2	2018/4/8	4627683B2B		
		Calumet-Bartlett Bend: River km 937.8–931.5	2018/4/12	47134D0F2A		
Wild	Male	Calumet-Bartlett Bend: River km 937.8–931.5	2018/4/12	470B110F50		
		Lower Plattsmouth Bend: River km 952.2–947.9	2018/4/7	462622242B		
		Lower Plattsmouth Bend: River km 952.2–947.9	2018/4/6	43449D6C1E		
		Tobacco Bend: River km 947.9–943.1	2018/4/4	4704510611		

## Data Availability

All raw sequencing reads were deposited into NCBI under BioProject PRJNA806960. The annotated sequencing files were uploaded to MG-RAST and are available in the MG-RAST Projects Sarah Sturgeon A-E.
